# Prosaposin Overexpression following Kainic Acid-Induced Neurotoxicity

**DOI:** 10.1371/journal.pone.0110534

**Published:** 2014-12-02

**Authors:** Hiroaki Nabeka, Keigo Uematsu, Hiroko Takechi, Tetsuya Shimokawa, Kimiko Yamamiya, Cheng Li, Takuya Doihara, Shouichiro Saito, Naoto Kobayashi, Seiji Matsuda

**Affiliations:** 1 Department of Anatomy and Embryology, Ehime University Graduate School of Medicine, Toon, Ehime, Japan; 2 Laboratory of Veterinary Anatomy, Faculty of Applied Biological Sciences, Gifu University, Yanagido, Gifu, Japan; 3 Medical Education Center, Ehime University Graduate School of Medicine, Toon, Ehime, Japan; Federal University of Rio de Janeiro, Brazil

## Abstract

Because excessive glutamate release is believed to play a pivotal role in numerous neuropathological disorders, such as ischemia or seizure, we aimed to investigate whether intrinsic prosaposin (PS), a neuroprotective factor when supplied exogenously *in vivo* or *in vitro*, is up-regulated after the excitotoxicity induced by kainic acid (KA), a glutamate analog. In the present study, PS immunoreactivity and its mRNA expression in the hippocampal and cortical neurons showed significant increases on day 3 after KA injection, and high PS levels were maintained even after 3 weeks. The increase in PS, but not saposins, detected by immunoblot analysis suggests that the increase in PS-like immunoreactivity after KA injection was not due to an increase in saposins as lysosomal enzymes after neuronal damage, but rather to an increase in PS as a neurotrophic factor to improve neuronal survival. Furthermore, several neurons with slender nuclei inside/outside of the pyramidal layer showed more intense PS mRNA expression than other pyramidal neurons. Based on the results from double immunostaining using anti-PS and anti-GABA antibodies, these neurons were shown to be GABAergic interneurons in the extra- and intra-pyramidal layers. In the cerebral cortex, several large neurons in the V layer showed very intense PS mRNA expression 3 days after KA injection. The choroid plexus showed intense PS mRNA expression even in the normal rat, and the intensity increased significantly after KA injection. The present study indicates that inhibitory interneurons as well as stimulated hippocampal pyramidal and cortical neurons synthesize PS for neuronal survival, and the choroid plexus is highly activated to synthesize PS, which may prevent neurons from excitotoxic neuronal damage. To the best of our knowledge, this is the first study that demonstrates axonal transport and increased production of neurotrophic factor PS after KA injection.

## Introduction

Prosaposin (PS) is the precursor protein of four small lysosomal glycoproteins, saposins A, B, C, and D [1], [2]. Each saposin activates specific lysosomal sphingolipid hydrolases, including cerebrosidase, ceramidase, sphingomyelinase, galactosidase and arylsulfatase A [3], [4]. Both saposins and PS are widely expressed in various tissues, although the brain, skeletal muscle and heart cells predominantly contain unprocessed PS rather than saposins [5]–[10]. In addition, unprocessed PS is found in various secretory fluids, such as seminal plasma, bile, pancreatic juice, human breast milk and cerebrospinal fluid [11], [12], and PS mRNA is strongly expressed in the choroid plexus [13].

In addition to its role as a saposin precursor, PS has been identified as a potent neurotrophic factor [14] and exists ubiquitously in nervous tissues [8], [15]. PS and prosaptide, a peptide containing the neurotrophic activity domain of PS, promote neurite outgrowth, elevate choline acetyltransferase activity in neuroblastoma cells [14] and prevent programmed cell death in cultured cerebral granule neurons [16], [17]. In cultured Schwann cells and oligodendrocytes, PS showed myelinotrophic activity that prevented cell death and increased myelin constituents [18], [19]. According to *in vivo* experiments, PS and amino acid 18-derived from PS facilitated sciatic nerve regeneration after transection [20] and rescued hippocampal CA1 neurons from lethal ischemic damage [21], [22] and dopaminergic neurons from MPTP-induced neurotoxicity [23].

Kainic acid (KA), a glutamate analogue, is a powerful neurotoxic agent [24] that stimulates excitatory neurotransmitter release [25]. Systemic KA injection induces neuronal degeneration in certain neuronal areas, including the hippocampus [26]–[30]. The nature of neuronal degeneration caused by systemic KA injection resembles some forms of ischemia [31] or epilepsy [32], and KA has been used to define the mechanisms of neurodegeneration and neuroprotection [33].

Although the PS receptors have been defined, after debate over the past two decades [34], the movement of intrinsic PS in injured, as well as normal, nervous tissue remains unclear. We have shown that intrinsic PS and its mRNA increase in the facial nerve nucleus after nerve transection [35], [36] and decrease in the brain of mdx mice [37]. In the present study we aimed to determine whether intrinsic PS is up-regulated in brain neurons and the choroid plexus after systemic KA injection.

## Materials and Methods

### Animals

Ten-week-old male Wistar rats (320–350 g) were used in this study. All animals were provided by CLEA-Japan (Kyoto) and housed at a constant temperature (22°C) under a 12∶12-h light: dark cycle and given food and water *ad libitum*. This study was carried out in strict accordance with the recommendations of the Guidelines of the Animal Care Committee of Ehime University. The protocol was approved by the Animal Care Committee of Ehime University (Permit Number: 05A261). All surgery was performed under sodium pentobarbital anesthesia, and all efforts were made to minimize suffering.

### Antibodies

**Figure 1:**
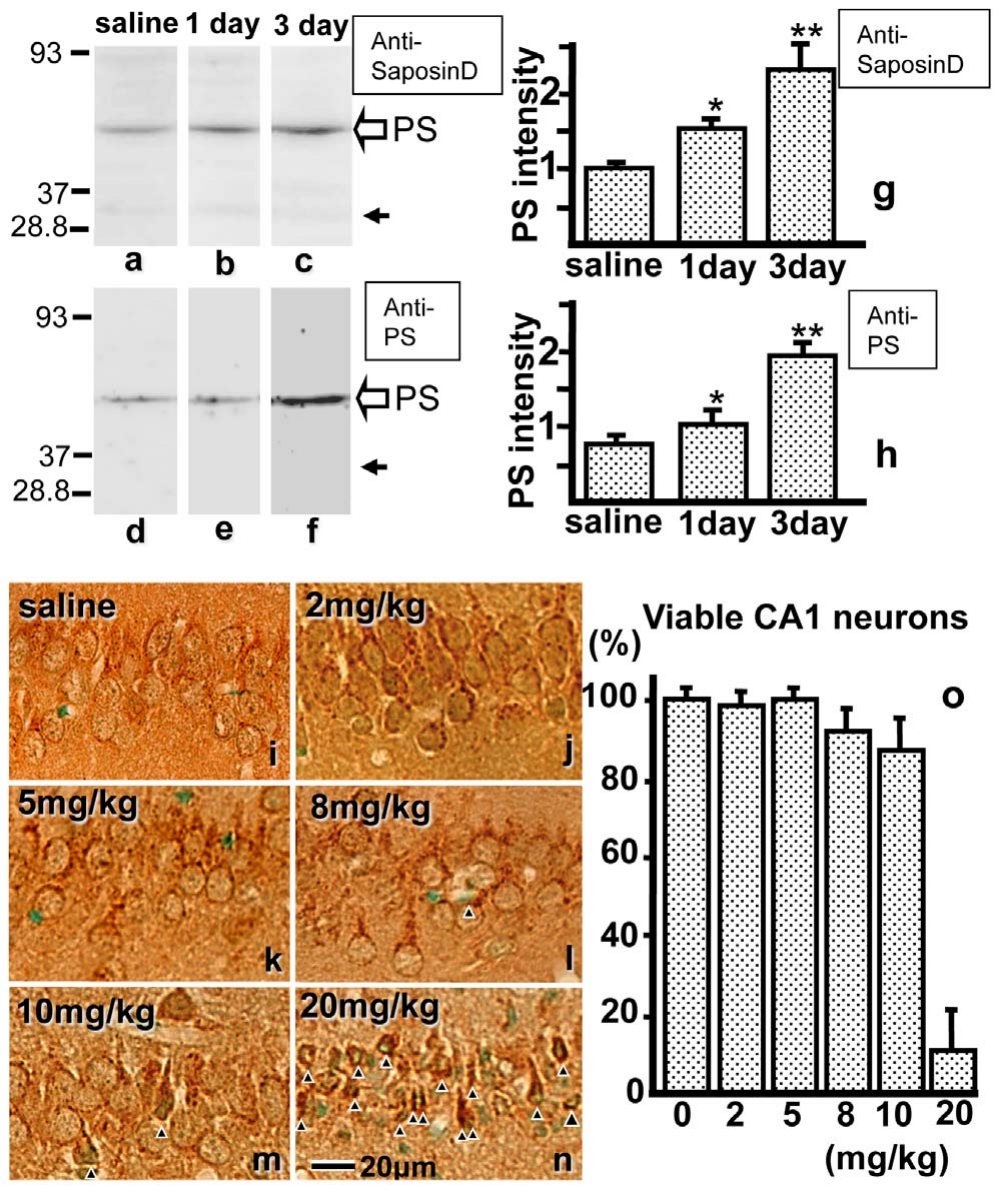
a–h: Crude hippocampal extracts from normal controls and from animals injected with kainic acid (KA) after 1 and 3 days were examined using anti-saposin D antiserum (a, b, c) and anti-PS IgG (d, e, f). When stained with anti-saposin D antiserum, an intense ∼69-kDa band (open arrow), which likely corresponds to prosaposin (PS), increased in intensity after KA treatment (b, c). Conversely, a faint ∼29-kDa band (closed arrow), which likely corresponds to di- or tri-saposin, did not change in intensity. When stained with anti-PS (d, e, f), the single band observed at ∼69 kDa (arrow), which likely corresponded to PS, increased in intensity after KA treatment. The intensities of the protein bands of PS were quantified using the NIH Image software (g, h). **i-n:** PS-IR in the hippocampal CA1 neurons from rats injected with saline (i), or 2 (j), 5 (k), 8 (l), 10 (m), or 20 (n) mg/kg KA at 7 days after injection. Arrowheads indicate damaged neurons. o: Pyramidal neurons with intact morphology along 1-mm linear length fields of hippocampal CA1 were counted, and the percentage of viable neurons in the hippocampal CA1 region after injection of saline or KA was calculated. Bar  = 20 µm.

Rabbit anti-saposin D antiserum was kindly provided by Professor A. Sano. Anti-PS IgG (0.1 µg/mL) was prepared by Medical and Biological Laboratories (Nakaku, Nagoya, Japan) [43]. From the amino acid sequence of rat PS (M19936; Collard et al. 1988), a synthetic oligopeptide corresponding to the proteolytic portion of PS (409- PKEPAPPKQPEEPKQSALRAHVPPQK-434), which did not encode saposins, was used to generate a rabbit polyclonal antibody against rat PS. Anti-saposin D antiserum reacted with saposin D and PS (Fig. 1a–c), and anti-PS IgG reacted with PS but not saposins (Fig. 1d–f).

### Preliminary study to determine the optimum KA dose

Rats (n = 3 per group) were anesthetized with an intraperitoneal injection of diethylether and clonazepam (0.2 mg/kg) as an anticonvulsant. After 10 min, the rats were reanesthetized with diethylether, and KA dissolved in normal saline was injected subcutaneously in varying amounts (0, 2, 5, 8, 10 and 20 mg/kg) to determine the optimal dose that can stimulate neurons but not kill them. After KA injection, the animals were housed at a constant temperature (22°C), as the effects of KA depend, at least in part, on body temperature.

Seven days after KA injection, each animal was anesthetized by intraperitoneal injection of chloral hydrate (10 mg/kg) and perfused transcardially, first with 50-mL saline and then with 300-mL 4% paraformaldehyde in 0.1 M phosphate buffer. The tissues were removed, cut into small pieces and post-fixed in the same solution for 4 h. The tissues were embedded in paraffin using conventional methods, sectioned and deparaffinized. Following a brief rinse in PBS, the sections were exposed for 2 h to blocking solution containing 5% normal swine serum (NSS), 5% bovine serum albumin (BSA) and 0.25% carrageenan in PBS. The sections were processed for immunohistochemistry with previously prepared anti-saposin D antiserum as the primary antibody at a 1∶1,000 dilution [5], [11]. The specificity of the anti-saposin D antiserum was verified using Western blot by a previous study [10]. The sections were then rinsed with PBS and incubated with biotinylated anti-rabbit IgG (DAKO, Denmark) for 30 min at room temperature. After rinsing with PBS, the sections were incubated overnight at 4°C with peroxidase-conjugated streptavidin (DAKO). Finally, the sections were rinsed with PBS, and the color reaction was developed using the diaminobenzidine (DAB) method. The CA1 region of animals injected with 2 or 5 mg/kg KA contained no damaged neurons; however, some damaged neurons were observed with 8 or 10 mg/kg KA, and many were found with 20 mg/kg KA. The viable neurons in a 1,000-µm linear length of the CA1 region were counted as described elsewhere [38], [39]. Based on the results, we determined the optimum dose of KA for this experiment to be 5 mg/kg. Control sections were incubated with pre-immune serum, and no specific staining was observed (data not shown) [9].

### Analysis of PS after KA injection using Western blot

The rats were anesthetized and injected with clonazepam (0.2 mg/kg), followed by 5 mg/kg KA as mentioned above. On days 1, 3, 7, 14 and 21 after KA injection, the hippocampi of the rats (n = 6 per group) were dissected and homogenized using a homogenizer (Taitec, Koshiya, Saitama, Japan) in ice-cold 50 mM Tris-HCl (pH 6.8) buffer containing 0.1 M glycerol, 50 mM sodium dodecyl sulfate (SDS) and 4% protease inhibitor cocktail (Roche Diagnostics, Minatoku, Tokyo, Japan). The total protein extract was centrifuged at 12,000×g for 15 min at 4°C, and the separated pellet was suspended in buffer in an equal volume as that of the supernatant. The solubilized proteins (50 µg) were separated by SDS-polyacrylamide gel electrophoresis (PAGE) on 10% polyacrylamide gels and transferred to a polyvinylidene difluoride membrane. The membrane was incubated with 5% BSA in TBS buffer (20 mM Tris-HCl, pH 7.4; 0.15 M NaCl) for 1 h at room temperature and then incubated with anti-saposin D serum (0.05 µg/mL) and anti-PS IgG (0.1 µg/mL) overnight at 4°C. The membrane was then washed with TBS-T buffer (20 mM Tris-HCl, pH 7.4, 0.15 M NaCl, 1% Tween 20) and incubated with peroxidase-labeled anti-rabbit IgG (0.6 µg/mL; Dako, Glostrup, Denmark) for 1 h at room temperature, followed by treatment with the detection solution for enhanced chemiluminescence (ImmunoStar; Wako, Osaka, Japan). The immunoreactive protein bands were visualized using an LAS-4000 luminescence image analyzer (GE Healthcare Japan, Japan).

### Analysis of PS after KA injection using immunofluorescence

The rats were anesthetized and injected with clonazepam (0.2 mg/kg) followed by 5 mg/kg KA as described above. On days 1, 3, 7, 14 and 21 after KA injection, the rats (n = 6 per group) were fixed transcardially, and their brains were embedded in paraffin. Immunofluorescence was used to compare the PS immunoreactivity. The sections were deparaffinized and treated with blocking solution as described above, then incubated overnight at 4°C in blocking solution containing rabbit anti-saposin D antiserum (1∶250) or anti-PS IgG. After washing with PBS, the sections were treated for 1 h at room temperature with Cy3-conjugated goat anti-rabbit IgG (1∶500; Rockland, Gilbertsville, PA, USA) for detection of saposin D. Some sections were double-stained with anti-MAP2 monoclonal antibody for dendrite detection, and rabbit anti-saposin D antiserum (1∶250) or anti-PS IgG, then treated with Cy3-conjugated goat anti-rabbit IgG (1∶500; Rockland, Gilbertsville, PA, USA) and Alexa Fluor 488 goat anti-mouse IgG (H+L) (1∶1000; Life Technologies, Carlsbad, CA, USA). The sections were then washed with PBS, mounted in Mowiol (Calbiochem, San Diego, CA, USA) and examined under a fluorescence microscope (BZ-8000; Keyence, Osaka, Japan). High-resolution confocal images were obtained using a Nikon A1 confocal microscope (Nikon, Tokyo, Japan) equipped with a 60× objective lens (Nikon). The relative intensity of PS-IR signals in the hippocampal CA1 region was examined using computer-assisted image analysis as described below.

### Double immunostaining of PS and GABA or of PS and Tau

Three days after KA injection, rat brains were fixed, embedded in paraffin and sectioned as described above, except that the fixation solution contained 0.5% glutaraldehyde as well as 4% paraformaldehyde. The deparaffinized sections were immunostained as mentioned above. After blocking, they were incubated in a solution containing rabbit anti-PS IgG and mouse anti-GABA monoclonal antibody (SWANT, Bellinzona, Switzerland), then in Cy3-conjugated goat anti-rabbit IgG (1∶500; Rockland), Alexa Fluor 488 goat anti-mouse IgG (H+L) (1∶1000; Life Technologies) and DAPI. The brains were then washed with PBS, mounted in Mowiol (Calbiochem) and examined under a fluorescence microscope (BZ-8000; Keyence). High-resolution confocal images were obtained using a Nikon A1 confocal microscope (Nikon, Tokyo, Japan) equipped with a 60× objective lens (Nikon).

For double staining of PS with Tau (an axon marker), the deparaffinized sections were incubated in a solution containing rabbit anti-PS IgG, goat anti-Tau IgG (American Res. Prod. Inc., Waltham, MA, USA) and DAPI. The sections were further stained with secondary antibodies and observed as described above.

### Analysis of PS mRNA after KA injection using in situ hybridization

The rats were anesthetized and injected with clonazepam (0.2 mg/kg) followed by 5 mg/kg KA as described above. On days 1, 3, 7, 14 and 21 after KA injection, the rats (n = 6 per group) were decapitated, the forebrains were frozen on dry ice and cut into 20-µm frontal sections using a cryostat. *In situ* hybridization to detect PS mRNA was performed as described previously [35], [40], [41]. Briefly, an antisense 36-mer oligonucleotide probe (PSA1: 5'-TTCATTACCCTAGACCCACAAG TAGGCGACTTCTGC -3′) complementary to bases 1704–1739 of rat PS mRNA [42] was synthesized. A sense oligonucleotide probe (PSS1) complementary to the bases of the PSA1 probe was also synthesized as a control. The frozen sections were fixed in 4% paraformaldehyde in 0.1 M PBS (pH 7.4) for 15 min, rinsed with 4× standard saline citrate (SSC; pH 7.4) and dehydrated using a graded ethanol series. The sections were then hybridized overnight at 41°C with the ^35^S-labeled antisense or sense probe at 1.0×10^7^ cpm/mL in hybridization buffer (50% formamide, 1% Denhardt's solution, 250 µg/mL tRNA, 0.1 g/mL dextran sulfate, 0.12 M PB, 0.02 mM/mL DTT in 4×SSC). After hybridization, the sections were rinsed three times with 1× SSC at 55°C for 20 min, dehydrated using a graded ethanol series, coated with NBT2 emulsion (Eastman Kodak Company, Rochester, NY, USA) and exposed for 3 weeks at 4°C. Finally, the sections were developed using a D-19 developer (Eastman Kodak) and observed under a light microscope. Controls for *in situ* hybridization using the sense probe, the antisense probe with a 100-fold excess of unlabeled antisense probe, or the antisense probe after RNase treatment showed no signal.

### Statistical analysis

The relative intensities of immunoreactivity in the immunoblot bands or immunohistochemistry and hybridization signals in the hippocampus were blindly examined using computer-assisted image analysis. Briefly, digital images of the central parts of CA1, CA3, CA4, and dentate gyrus (DG) were obtained using a fluorescence microscope and light-field microscope equipped with a digital camera. The images were obtained under the same magnification and voltage in order to stabilize brightness. The average gray value of all pixels in each image was determined using NIH 1.56 software (public domain software by Dr. Steve Barrett). Then the ratio of the gray values obtained from the image was calculated. The statistical significance of the ratios was examined by one-way analysis of variance (ANOVA) and *post hoc* Fisher's PLSD tests using the program StatView (Abacus Concepts Inc., Berkeley, CA, USA).

## Results

### Western blot

Immunoblotting of the hippocampus with an antibody against saposin D showed two bands at approximately 69 and 30 kDa; these bands likely corresponded to PS and di- or trisaposin, respectively (Fig. 1a–c, g). The faint di- or trisaposin band did not change in intensity after KA treatment regardless of the strong increase in the PS band. Immunoblotting of the hippocampus using the specific antibody against PS showed only one band at approximately 69 kDa and showed a strong PS increase after KA treatment (Fig. 1d–f, h). Immunoblotting of the hippocampus using antiserum against saposin D or an antibody against PS showed a clear increase in PS after KA injection (Fig. 1a–h), but no clear saposin-specific bands, as has been reported previously in the spleen and other tissues [5], [43].

### Change in PS-like immunoreactivity (PS-IR)

**Figure 2:**
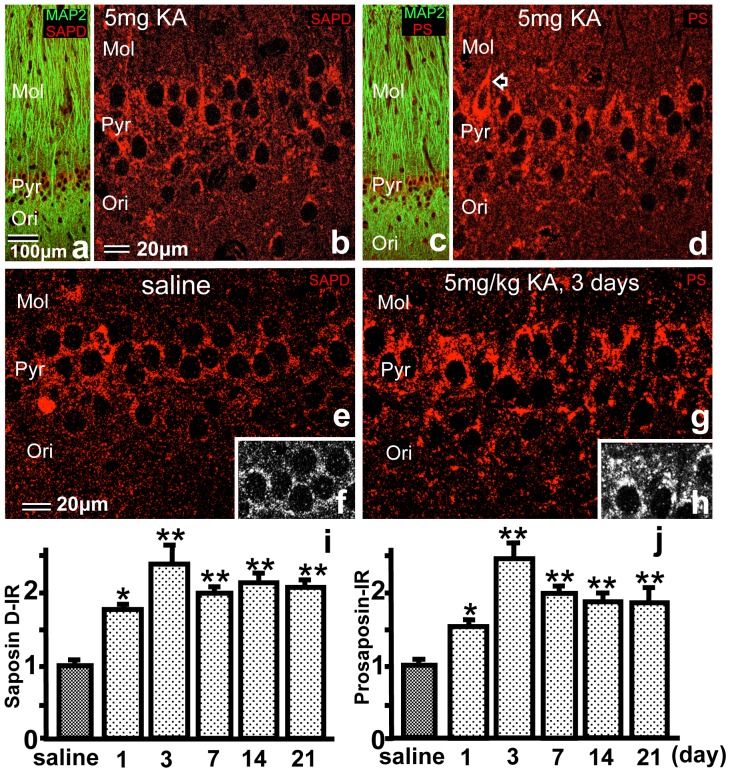
a–d: Light micrographs of the hippocampal CA1 neurons stained with anti-saposin D (a, b) or anti-PS IgG (c, d) showing the PS-IR 3 days after 5 mg/kg KA injection. The dendritic structure of the CA1 region was stained green with an anti-Map II antibody (a, c). b and d show higher-magnification images of the pyramidal layer (Pyr) shown in a and c, respectively. The arrow indicates an interneuron with a slender nucleus and very intense PS-IR cytoplasm (d). **e-h:** Light micrographs of the hippocampal CA1 neurons of normal controls (e) and animals 3 days after 5 mg/kg KA injection (g). f and h are black-and-white images of the pyramidal layer (Pyr) shown in figures e and g, respectively, which were used for the NIH Image analysis. (i, j) Bar  = 100 µm (a, c) and 200 µm (b, d–h). **i, j:** The ratio of PS-IR in the hippocampal CA1 region after injection of KA or saline. PS-IR was semi-quantified using NIH image analysis. The ratio of PS-IR was significantly higher on days 1, 3, 7, 14 and 21 after KA injection compared with controls injected with saline (^*^ *p*<0.05, ^**^ *p*<0.01). Mol, stratum moleculare; Rad, stratum radiatum; Ori, stratum orience.

PS-IR in the hippocampal CA1 using the anti-saposin D antiserum showed similar staining patterns as shown previously [9]; PS-IR was visualized as dot-like in the organelles and as diffuse in the cytoplasm or cell membrane of nerve cell bodies and their large dendrites, but not in their nuclei (Fig. 1, 2). PS-IR was observed in the control animals (Fig. 1i, 2e), but stronger PS-IR was observed in the hippocampus of KA-injected animals 3 days after KA injection (Fig. 1j–n, 2g). Conversely, in the DAB-stained sections, many damaged neurons were observed as dark and shrunken in the CA1 of animals injected with 20 mg/kg KA (Fig. 1n), and similar neurons were observed after 8 or 10 mg/kg injection of KA, as well as PS-IR neurons (Fig. 1l, m). Although DAB reactivity increased after KA injection (Fig. 1j–n), artificial DAB reactivity increased in the sections containing injured neurons (Fig. 1m, n). The healthy neurons were counted and were approximately 100% in the hippocampal sections of normal controls and animals injected with 2 or 5 mg/kg KA and below 90% in animals injected with 8 or 10 mg/kg, but were below 10% with 20 mg/kg KA (Fig. 1n, o). From these results, we determined the maximal dose that did not cause any neuronal cell death was 5 mg/kg.

Immunofluorescent light micrographs of the hippocampal CA1 region showed similar dot-like PS-IR with the DAB staining. The immunofluorescent signals were observed as dot-like particles around nuclei or in the large dendrites both in normal controls and KA-injected animals (Fig. 2). Although the distribution patterns of saposin-D and PS were almost similar, their intensities were higher in the sections stained with anti-PS antibody than in sections stained with anti-saposin-D antiserum (Fig. 2b, d), which may be due to the differences between antiserum and purified IgG. For morphometrical analysis of the chronological change in PS-IR after KA injection, the red PS-IR fluorescence (Fig.2e, g) was changed to a black-and-white image (Fig. 2f, h), and the intensity was analyzed using NIH image software (Fig. 2i, j). The PS-IR increased on day 1 after KA injection, peaked on days 3 and 7 and remained significantly elevated until day 21 (Fig. 2i, j). Other areas such as CA3, CA4 and DG also showed similar patterns.

In the KA-injected animals, especially 3 days after KA injection, some cells with intense PS-IR were observed inside/outside of the pyramidal (Pyr) layer, and these cell bodies and nuclei were more slender than those of Pyr neurons, but larger than those of glial cells (Fig. 2d).

### Expression of PS mRNA in the hippocampus after KA injection

**Figure 3:**
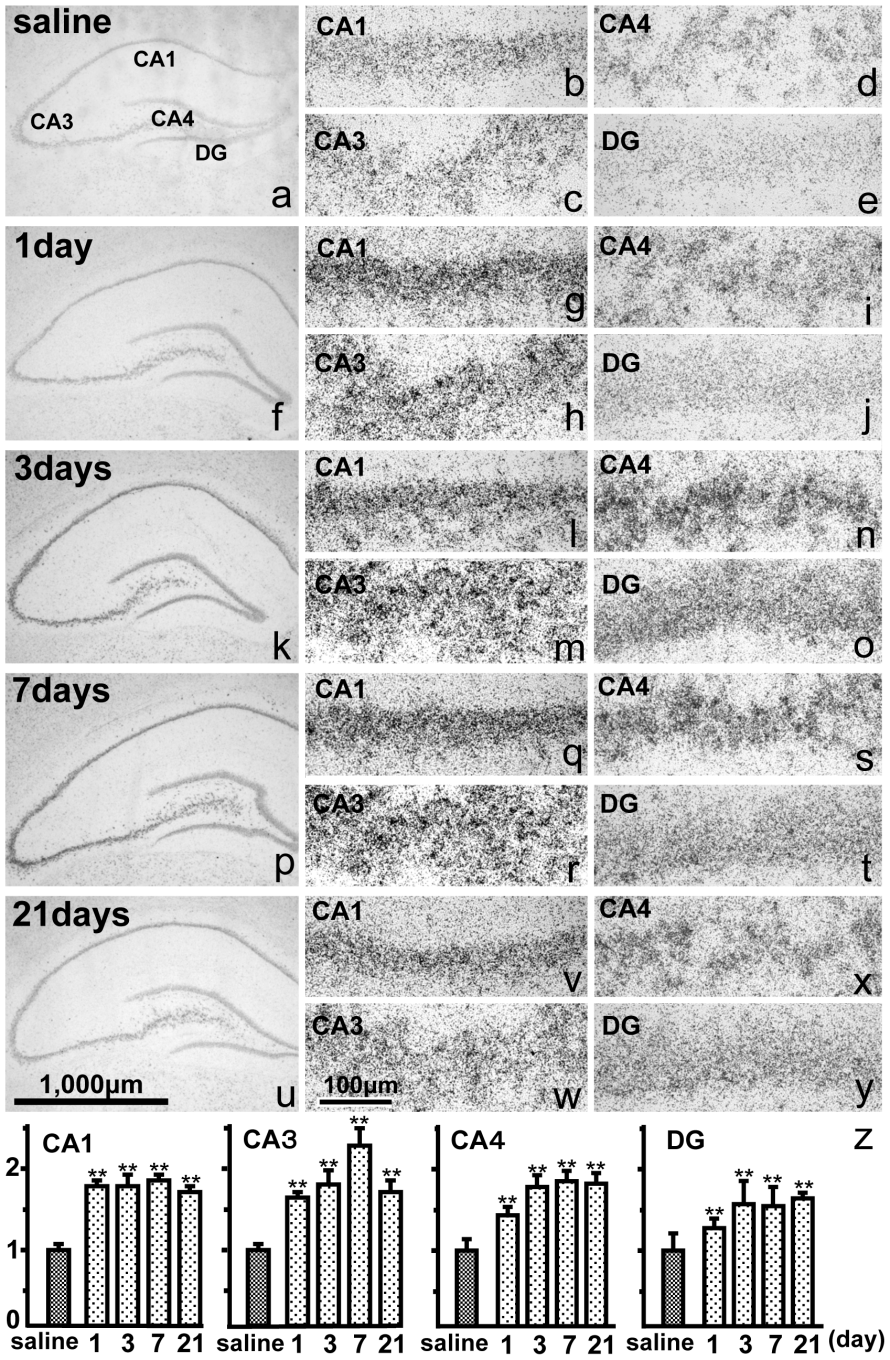
a–y: *In situ* hybridization of the hippocampus showing PS mRNA after saline injection (a–e), or 1 (f–j), 3 (k–o), 7 (p–t) and 21 days (u–y) after KA injection into the CA1 (b, g, l, q, v), CA3 (c, h, m, r, w), CA4 (d, i, n, s, x), and dentate gyrus (DG, e, j, o, t, y). PS mRNA expression increased after KA injection compared with saline injection. Bar  = 1,000 µm (a, f, k, p, u) and 100 µm (b–e, g–j, l–o, q–t, v–y). z: The ratio of the PS mRNA signals in the hippocampal CA1, CA3, CA4, and DG regions after the injection of KA or saline. The intensity of PS mRNA, semi-quantified as the ratio of reaction products, significantly increased on days 1, 3, 7 and 21 after KA injection compared with saline injection. The data are presented as means ± standard error (S.E.). **p<0.01; significantly different from control.

The expression of PS mRNA in the hippocampus after KA injection was examined by *in situ* hybridization using the antisense probe PS-AS1, which detects both alternatively spliced forms of PS mRNA [36]. In the saline-injected control animals, weak hybridization signals were observed in the neuronal cell layers of hippocampal CA1, CA2, CA3, CA4 areas and DG (Fig. 3a–e). One day after KA injection, hybridization signals abruptly intensified in all hippocampal areas (Fig. 3f–j), peaked on days 3 and 7 and remained significantly elevated until day 21 (Fig. 3k–y).

The hybridization signals as calculated using image analysis increased more than 150% of the control group signals and showed a statistically significant difference between the control and KA-injected groups (one-way ANOVA, *p*<0.0001; Fig. 3z). *Post hoc* Fisher's PLSD tests demonstrated that the ratios on days 1, 3, 7, 10 and 21 were significantly different from those of the control animals (*p*<0.01) (Fig.3z).

### Expression of PS mRNA in the interneurons

**Figure 4:**
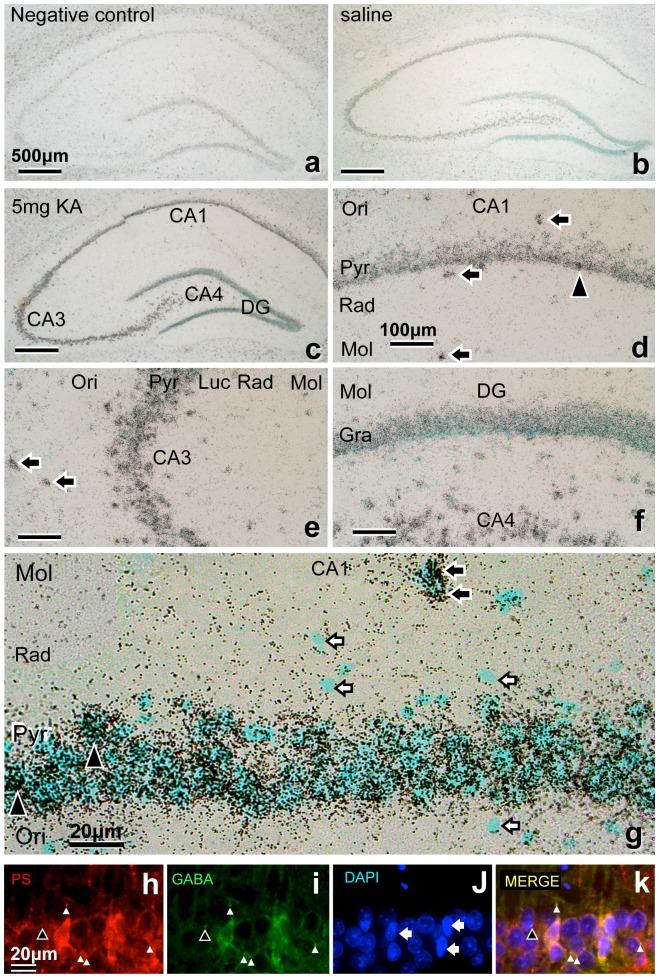
a–g: *In situ* hybridization of the hippocampus showing PS mRNA expression. The hybridization signals are shown as tiny black dots, and the nuclei were stained bluish green with methyl green. The section stained with the sense probe showed only faint hybridization signals (a: negative control). Compared with normal controls (saline injection) (b), the antisense probe signals in all hippocampal Pyr or granular (Gra) layers were significantly more intense 3 days after KA injection (c–g). Closed arrows indicate the intense interneurons in the extra-Pyr layer (d, e, g), and closed arrowheads indicate the interneurons in the intra-Pyr layer (g) with slender nuclei. Open arrows indicate smaller glial nuclei (g). **h–k**: Double immunofluorescence light micrographs of the rat hippocampal CA1 region 3 days after KA injection stained with anti-PS and anti-GABA. PS are stained with anti-PS (h: red), GABA with anti-GABA (i: green), nuclei are stained with DAPI (j: blue), and merged images are shown in (k). White arrowheads indicate double-stained terminals, and the black arrowhead indicates single-stained GABA terminals. Arrows indicate slender nuclei of GABA neurons. Bars  = 500 µm (a–c), 100 µm (d–f), and 20 µm (g–k).

The section stained with sense oligonucleotide probe (PSS1) showed only faint hybridization signals (Fig. 4a). The *in situ* hybridized hippocampal sections were counterstained with methyl green to visualize the nuclei (Fig. 4a–g). Compared with the normal control animals (Fig. 4b), the hybridization signals of the antisense probe PSA1 in all hippocampal areas were significantly strong after KA injection (Fig. 4c–g). The hybridization signals localized mainly in the Pyr layers or granular (Gra) layer, but some strong signals were observed outside of these layers, such as in the stratum radiatum and moleculare or the upper portion of the stratum oriens (Fig. 4d, e, g). Based on their size and localization, these cells appeared to be interneurons and not glial cells.

### Double immunofluorescence staining of PS and GABA

To clarify which types of cells strongly express PS after KA injection, the colocalization of PS and GABA was examined by double immunofluorescence staining of the hippocampus 3 days after KA injection. The intensities of PS and GABA immunoreactivity in the interneurons varied, but were greater overall than in the CA1 Pyr neurons (Fig. 4h–k).

### Double immunofluorescence staining of PS and Tau

**Figure 5:**
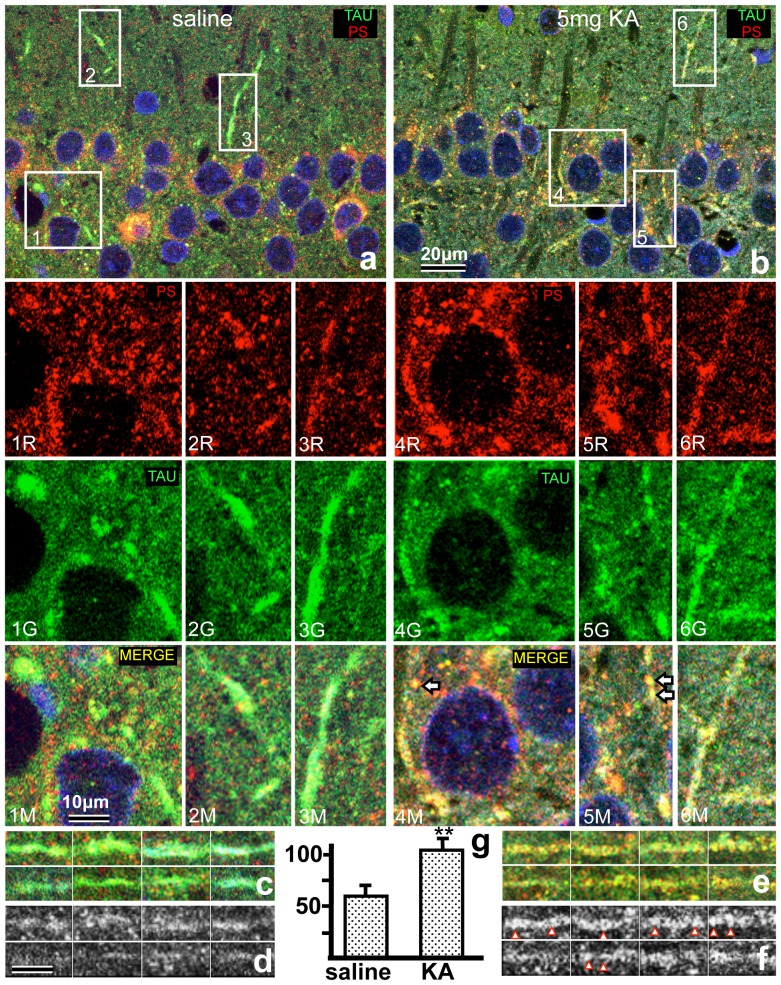
a, b: Double immunofluorescence light micrographs of the rat hippocampal CA1 regions of normal control (a) and animals 3 days after KA injection (b), stained with anti-PS and anti-Tau. The rectangles 1–6 in figures (a) and (b) are shown at a higher magnification in the lower column. Nuclei are stained with DAPI (blue), PS stained with anti-PS (1–6R: red), axons and axon terminals with anti-Tau (1–6G: green) and merged images (1–6M). Arrows indicate the double-stained axon terminals containing PS. **c–f:** Double immunofluorescence light micrographs of Tau-positive axons in the CA1 regions of normal control (c) and experimental animals 3 days after KA injection (e), stained with anti-PS and anti-Tau. Panels d and f are black-and-white images of the PS-IR shown in c and e, respectively, for the NIH Image analysis (g). Note that the PS-IR granules (arrowheads) in the Tau-positive axons in the KA-injected animals are larger than those in the control. **g:** The ratio of PS-IR in the Tau-positive axons around the Pyr-layer of the CA1 region after injection of KA or saline. The ratio of PS-IR was significantly higher after KA injection compared with controls injected with saline (^**^ *p*<0.01). Bars  = 20 µm (a, b) and 10 µm (1R–6M, c–f).

To show the increase of PS in axons after KA injection, the colocalization of PS and Tau was examined by double immunofluorescence staining of the hippocampus 3 days after KA injection. The intensity of PS in the Tau-positive structures, such as transient axons or terminal boutons around Pyr neurons in the hippocampal CA1, increased significantly in KA-injected animals compared with controls (Fig. 5). The percentages of PS-immunoreactive granules in the Tau-positive terminal boutons in the CA1 region of KA-injected or normal animals were approximately 90% and 60%, respectively. For morphometric analysis, the red PS-IR fluorescence in the Tau-positive axons (Fig. 5c and e) was transformed to a black-and-white image (Fig. 5d and f), and the intensity was analyzed using the NIH Image software. PS-IR in the axons significantly increased after KA-injection (Fig. 5g); in particular, the PS-IR granules after KA injection were larger than those after saline injection (Fig. 5f). The axon terminals around the Pyr neurons also showed double immunofluorescence for PS and GABA (Fig. 5-4M, 5M).

### PS mRNA expression in the cerebral cortex

**Figure 6:**
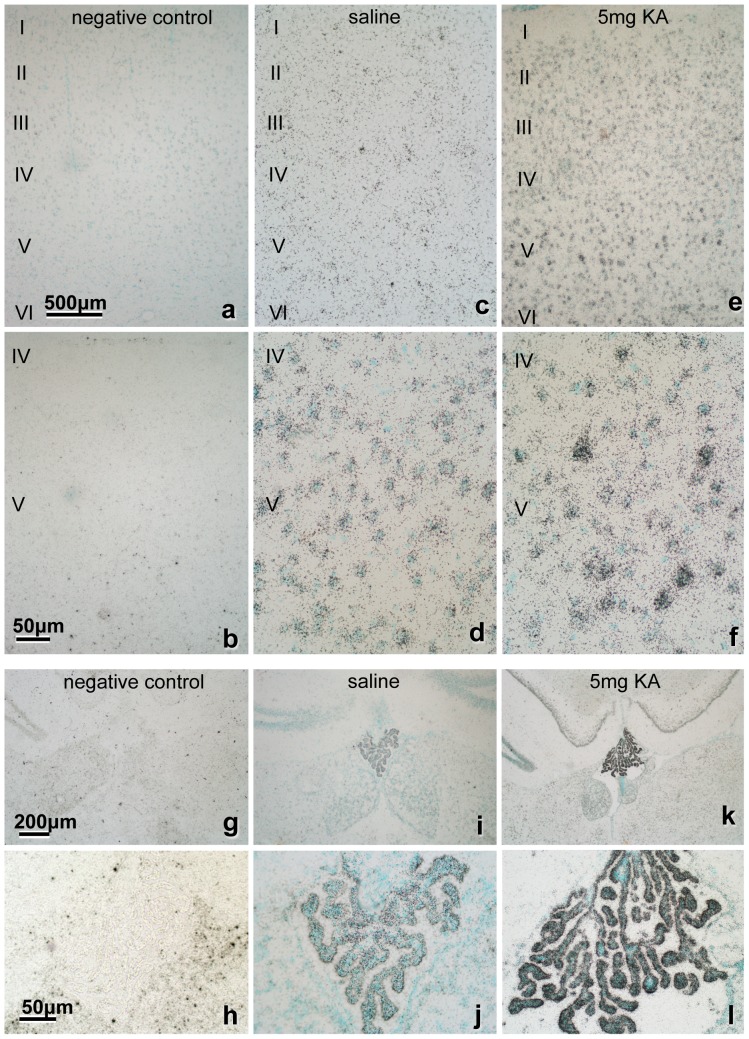
*In situ* hybridization of the cerebral cortex showing PS mRNA expression. The section stained with sense probe showed only faint hybridization signals (a, b, g, h). Compared with normal control (c, d, I, j), the signals of the antisense probe in all layers of cortex (e, f) and the choroid plexus (k, l) were significantly more intense after KA injection. Notably, the large Pyr neurons (f) and the choroid plexus (k, l) show very intense signals. Bar  = 500 µm (a, c, e), 50 µm (b, d, f, h, j, l) and 200 µm (g, i, k).

PS mRNA expression in the cerebral cortex was examined using *in situ* hybridization, similar to the method used for the hippocampus. In the saline-injected control animals, weak hybridization signals were observed in the neuronal cells in the six cortical layers (Fig. 6c, d). Conversely, 3 days after KA injection, hybridization signals abruptly intensified in almost all neurons, especially in the large Pyr neurons of layer V (Fig. 6e, f), peaked on days 3 or 7 and remained significantly elevated until day 21.

### PS mRNA expression in the choroid plexus

PS mRNA expression in the choroid plexus of saline-injected control animals was strong (Fig. 6i–j). However, 3 days after KA injection, the hybridization signal was very strong (Fig. 6k, l), peaked on days 3 or 7 and remained significantly elevated until day 21.

## Discussion

In the present study we aimed to investigate whether intrinsic PS was up-regulated in brain neurons and the choroid plexus after systemic KA injection. An increase in PS, but not saposins, in the brain was detected by immunoblot analysis. Stimulated neurons synthesize PS for survival, inhibitory interneurons transport PS and may secrete PS around hippocampal pyramidal neurons, and the choroid plexus highly synthesizes PS, which may protect brain neurons from excitotoxic damage.

The anti-saposin D serum does not react with the other saposins A, B, or C, but it does react with PS. As previously reported, two bands were observed at approximately 39 and 66 kDa in Western blot analyses of hippocampal tissue using anti-saposin D serum [9]. The molecular weight of PS is 65–70 kDa [44], whereas saposin is 12–16 kDa [4], [45]. The major proteolytic pathway of PS has been reported to begin with cleavage of saposin A from PS (tetrasaposin) and progress from B-C-D trisaposin to B-C and C-D disaposins and finally to monosaposin [46]. As quantified using the NIH Image software, the intensities of faint 39-kDa protein bands were less than 8% of those of the strongest 66-kDa bands. Therefore, in the present study, the strongest 66-kDa band represented an unprocessed precursor form of PS, while the faint 39-kDa band is likely a partially processed form of PS, such as di- or tri-saposin. These Western blot results suggest that the immunoreactions observed in the present study, both with anti-saposin D antiserum and anti-PS IgG, were mainly PS-specific. The results also confirmed that the increase in PS-like immunoreactivity after KA injection was not due to the increase in saposins as lysosomal enzymes after neuronal damage, but rather to the increase in PS as a neurotrophic factor.

An increase in PS mRNA expression was documented in ischemic rat brain injury. Yokota *et al*. reported significantly increased PS mRNA levels in the rat hippocampus 6 and 24 h after transient forebrain ischemia induced by the four-vessel occlusion method [47]. Hiraiwa *et al*. found significantly increased PS mRNA level in the brain 5–10 days after transient focal cerebral ischemia caused by occlusion of the middle cerebral artery [48]. A similar increase in PS mRNA expression was detected in the present study. Two alternative splicing forms of rat PS mRNA exist: Pro+9 mRNA and Pro+0 mRNA [48], and we analyzed PS mRNA expression in the hippocampus after KA injection using an antisense 36-mer oligonucleotide probe that recognized both types of PS [36]. PS mRNA expression increased significantly on day 1 and peaked on day 3 or 7 after KA injection; PS expression was significantly elevated even after 3 weeks (Fig. 3). The increased expression of PS mRNA for a relatively long time, as observed in the present study, contributes to the prevention of apoptosis in the damaged neurons. Therefore, this KA injection model not only mimicked the ischemic model but was also shown to be a good ischemic tolerance model of the increase in intrinsic neurotrophic factors.

Intrinsic PS-IR increased in facial motoneurons 3 days after facial nerve transection, decreased on day 7, began to increase gradually on day 14 and then reached another peak on day 21 [35]. PS mRNA increased after 3 days, peaked at 5 days, and remained elevated until 28 days after facial nerve transection [36]. These results indicated a very complex and dynamic pattern of PS expression in the facial motoneurons after facial nerve injury. In the present study, both PS-IR and PS mRNA peaked at 3 days after KA injection. The difference in expression pattern may be due to the difference of injury or neuron type.

Freund and Buzsaki proposed that the term hippocampal “interneuron” should be synonymous with “GABAergic with no principal cell” [49]. In the present study, interneurons outside of the Pyr layer were easily defined, and those inside the Pyr layer were defined as slender cell bodies and nuclei (Fig. 2d). Our results showed that PS mRNA signals in the interneurons increased abruptly 1 day after KA injection (Fig. 3), and those of PS-IR increased after 1 day and peaked 3 days after KA injection (Fig. 4). These results indicate that KA injection stimulated the interneurons more strongly than the Pyr neurons to produce PS and raise the question of the purpose of the increase in PS in interneurons.

Liang *et al*. reported that the amplitudes of evoked inhibitory postsynaptic currents were increased significantly 12 h after ischemia and then returned to control levels 24 h following reperfusion with voltage clamp recording [50]. They suggested that this transient enhancement of inhibitory neurotransmission might temporarily protect CA1 Pyr neurons and delay neuronal death after cerebral ischemia. Therefore, transient enhancement of inhibitory neurons might protect neurons by inhibiting Pyr neurons. Similarly, PS released from the axonal terminals of interneurons around the Pyr neurons also might protect the Pyr neurons. Accordingly, the PS-IR in the bouton-like structures around the Pyr neurons was strongly increased (Fig. 5–4M, 5M). In conclusion, the interneurons might protect Pyr neurons not only with inhibitory neurotransmission but also with PS release.

The reason why the Pyr neurons in the layer V of the cerebral cortex showed intense PS mRNA expression is unclear. Heggli *et al*. reported that the most pronounced effect of systemic injection of KA (12 mg/kg) induces necrosis and neuronal degeneration in the piriform cortex, amygdaloid complex, hippocampus and septum, but with no mention about the cerebral cortex [29]. In the present study, almost all neurons in the cerebral cortex showed increased PS mRNA expression after KA injection (Fig. 6e). In particular, layer-V Pyr neurons (Fig. 6f), which have a wide dendritic area that receives intense stimulation from many neurons following KA injection, produce a large amount of PS.

PS mRNA expression in the choroid plexus of normal control animals was strong (Fig. 6i–j), as reported previously [13]. This observation is reinforced by the finding that cerebrospinal fluid in normal animals contains a significant amount of PS [11]. Furthermore, 3 days after KA injection, the hybridization signal in the choroid plexus was very strong (Fig. 6k, l). These results indicate that PS is produced in the choroid plexus and may be secreted into the cerebrospinal fluid after direct or indirect KA stimulation to the choroid plexus.
